# DNA methylation of hepatic iron sensing genes and the regulation of hepcidin expression

**DOI:** 10.1371/journal.pone.0197863

**Published:** 2018-05-17

**Authors:** Paul A. Sharp, Rachel Clarkson, Ahmed Hussain, Robert J. Weeks, Ian M. Morison

**Affiliations:** 1 Department of Nutritional Sciences, School of Life Course Sciences, King’s College London, London, United Kingdom; 2 Department of Pathology, Dunedin School of Medicine, University of Otago, Dunedin, New Zealand; Lady Davis Institute for Medical Research, CANADA

## Abstract

Production of the iron regulatory peptide hepcidin is tightly controlled by a network of proteins in hepatocytes that sense levels of iron in the circulation (as diferric-transferrin) and in tissues (in ferritin). Human studies show high variability in the normal range of serum hepcidin levels. We have postulated that this may, in part, be related to inter-individual variability in the expression of genes in the iron sensing pathway, potentially governed by epigenetic factors. Here, we have investigated whether genes encoding hepatic iron sensing proteins and hepcidin are regulated by DNA methylation. Experiments were performed on two human hepatoma cell lines, HepG2 cells and Huh7 cells. Basal expression of *TFR2* and *HAMP* was significantly lower in Huh7 cells compared with HepG2 cells. Analysis of bisulphite-converted DNA from Huh7 cells revealed partial methylation of *TFR2* (alpha transcript), which could result in gene silencing. Demethylation using 5-aza-2’-deoxycitidine (AZA) increased *TFR2* mRNA expression in Huh7. PCR analysis of bisulphite-converted *HAMP* promoter DNA, using methylation-specific primers, revealed no differences between cell lines. However, *HAMP* mRNA expression in Huh7 was increased by AZA treatment, suggesting that methylation of one or more iron sensing genes may indirectly influence *HAMP* expression. Our study provides evidence that DNA methylation might control expression of *HAMP* and other hepatic iron sensing genes, and indicates that epigenetic influences on iron homeostasis warrant further investigation.

## Introduction

Iron homeostasis is maintained by a network of proteins in hepatocytes, which sense changes in circulating and cellular levels of iron. Downstream signalling from these sensors leads to the regulated production of hepcidin (reviewed in [1]). Once released from hepatocytes, serum hepcidin acts as a negative regulator of iron export from a number of cell types, including enterocytes and macrophages, by decreasing expression of the iron transport protein ferroportin [2–5]. In addition, in enterocytes, hepcidin limits iron absorption from diet through down-regulation of DMT1 [5–8].

In hepatocytes, increased cellular iron activates the bone morphogenetic protein (BMP) signalling pathway, which encompasses the ligand BMP6, BMP receptors and the co-receptor hemojuvelin (HJV), and induces the production of hepcidin via the SMAD signalling pathway [9,10]. The protein network which senses changes in serum iron levels (detected as changes in transferrin (Tf) saturation) and translates this information to produce appropriate levels of hepcidin is complex. At low Tf saturation levels, the hereditary haemochromatosis protein HFE and transferrin receptor 1 (TfR1) exist as a complex on the plasma membrane. However, at increased saturation levels, diferric-Tf competes with HFE for binding to TfR1. This causes HFE to dissociate from TfR1 and bind to a second transferrin receptor (TfR2). HFE/TfR2/diferric-Tf binding initiates an iron sensing pathway leading to increased production of hepcidin [11].

The *TFR2* gene contains two promoter regions and encodes two receptor isoforms. *TFR2*-alpha transcript encodes the full length receptor and is expressed on the cell surface of hepatocytes, erythroid progenitors and peripheral blood mononuclear cells [12]. The *TFR2*-beta transcript is a truncated form which lacks the membrane spanning domain and is highly expressed in spleen, brain and heart [12]. Its physiological role is unclear, but may be involved in regulation of ferroportin in splenic macrophages [13]. The involvement of each of the *TFR2* transcripts in iron sensing and the regulation of hepcidin production has not been studied previously and we have addressed this in our current work.

Mutations in *TFR2* give rise to type III haemochromatosis [14], which is characterised by liver iron loading and inappropriately low hepcidin levels [15,16]. Furthermore, evidence from knockout studies in mice demonstrates that deletion of *TFR2* [17,18], *HFE* [17,18], *HFE2* (*HJV*) [19,20], or *BMP6* [10] leads to inappropriately low *HAMP* (the gene encoding hepcidin) expression and liver iron loading. Taken together this suggests that each of these iron sensing elements has a specific role to play in the appropriate production of hepcidin. Interestingly, while the HFE/TfR2 and BMP signalling pathways regulating *HAMP* expression may operate independently of each other [21–23], there is also evidence for interaction between these sensing networks [17,24].

In addition to modulation by iron, *HAMP* expression is regulated by a variety of other signals including adipokines [25], pro-inflammatory cytokines [26–29], and hypoxia [30–34]. The normal range of serum hepcidin levels in the healthy population is highly heterogeneous [35–37]. While age and gender are significant factors in this distribution, there is still considerable variability (by as much as 50-fold) within population groups. The basis for this is unclear, but it could be related to the non-iron factors identified above (cytokine levels, etc.) or to inter-individual variability in the expression of genes in the iron sensing pathway. Gene expression at an individual level may be controlled by a number of genetic or epigenetic factors. One possible mechanism is through DNA methylation, which occurs at CG sites, resulting in the formation of 5-methylcytosine. CpG-rich regions, known as CpG islands, often occur within the promoter regions of genes, and when methylated result in the repression of gene expression [38]. In this study we have explored ENCODE DNA methylation data to identify putative methylation target sites in the promoter regions of iron sensing genes and *HAMP*, and assessed the effects of demethylation on mRNA expression of these genes in human hepatoma cell lines.

## Materials & methods

### Cell culture

HepG2 (ATCC) and Huh7 (gift from Prof. S. Srai, UCL) hepatoma cells were cultured in Dulbecco’s modified Eagle medium containing 10% fetal bovine serum, 100 units/ml penicillin, 100 μg/ml streptomycin, 2 mM L-glutamine and 1% non-essential amino acid solution (all purchased from Thermo Fisher Scientific, UK). For experiments, cells were seeded into 6-well plates at a density of 1 x 10^5^ cells/cm^2^. In some experiments cells were treated with 5-aza-2’-deoxycitidine (AZA, 5 μM) for 72 hours; medium containing AZA was replaced every 24 hours.

### Quantitative real-time (qRT)-PCR

RNA isolated from hepatic cells was converted to cDNA using a High Capacity cDNA Reverse Transcription Kit (Thermo Fisher Scientific). Expression of *TFR2* alpha transcript, *TFR2* beta transcript, *HAMP*, *HFE*, *BMP6*, *HFE2* (*HJV*) and *B2M* (reference gene) was analysed using Fast SYBR green Master Mix (Thermo Fisher Scientific) and a ABI Prism 7500 FAST sequence detection system (Applied Biosystems). Primer sequences are given in Table 1. Data were analysed using the 2^-ΔΔCt^ method [39].

**Table 1:** PCR primers sequences.

Primer	Forward sequence	Reverse Sequence
B2M RT	CCA CTG AAA AAG ATG AGT ATG CCT	CCA ATC CAA ATG CGG CAT CTT CA
HAMP RT	CTG CAA CCC CAG GAC AGA G	GGA ATA AAT AAG GAA GGG AGG GG
TFR2-α RT	GTC AGT GAG GAT GTC AA	CCA CAC GTG GTC CAG CTT CT
TFR2-β RT	CCA GAA AAG TCC CCA CCT C	TGC TCT CCG ACC TTC CC
HFE RT	AGA ACA GGG CCT ACC TGG AG	TGT GTC ACC TTC ACC AAA GG
HFE2 (HJV) RT	GGA GCT TGG CCT CTA CTG GA	ATG GTG AGC TTC CGG GTG
BMP6 RT	CCG TGT AGT ATG GGC CTC AGA	TCA CAA CCC ACA GAT TGC TAG T
TFR2-α BIS	GGG GGT TGA GGG ATT AGA GAA	CCA AAA CTA TAC CCC CAC CCT TAA AA
HAMP Met	TTT TGT TTT CGT TTA TTT TTT TCG T	AAA CTC AAT ACC ATC GTA CCG TC
HAMP Unmet	TTT TGT TTT TGT TTA TTT TTT TTG T	AAA AAC TCA ATA CCA TCA TAC CAT

RT = qRT-PCR primer; BIS = primers for bisulphite-converted DNA; Met = specific primers for methylated bisulphite-converted DNA; Unmet = specific primers for unmethylated bisulphite-converted DNA.

### Methylation PCR

DNA from HepG2 and Huh7 cells was subject to bisulphite conversion using EZ DNA Methylation-Gold™ kit (Zymo Research) according to the manufacturer’s instructions. For analysis of the *TFR2* alpha promoter, a 246 bp fragment containing the whole of exon 1, plus the 5’ flanking region and intron 1 (base pairs -154 to +92, relative to the translation start site), was amplified using bisulphite-specific primers (Table 1, designed using MethPrimer; www.urogene.org/cgi-bin/methprimer/methprimer.cgi). The amplicon contained a ClaI restriction digest site (ATCGAT) which allowed the PCR product to be cut into two fragments (138 and 108 bp, respectively) if the CpG within the restriction enzyme recognition site was methylated. Aliquots of ClaI-digested and undigested PCR products were resolved on 2% agarose-ethidium bromide gels. Further aliquots of the full amplicon were cloned into *E*. *coli* using a TOPO TA Cloning Kit (Invitrogen). Positive colonies were selected and DNA extracted and sequenced (ABI 3730xl DNA Analyser).

For analysis of the *HAMP* promoter, no suitable bisulphite-specific primers could be designed. Instead we designed methylation-specific primers (Table 1) that would bind to either the predicated methylated or unmethylated DNA sequences within the bisulphite-converted *HAMP* promoter. Primers targeted identical regions in the *HAMP* promoter, and differed only at two C/T bases in each of the forward and reverse primers. Analysis was carried out using qRT-PCR.

### Statistics

Data are presented as mean ± S.E.M. Data were analysed using Mann-Whitney tests and differences with p < 0.05 considered statistically significant. Linear regression analysis was performed using Sigmaplot (version 13, Systat Software Inc., UK).

## Results

**Fig 1:**
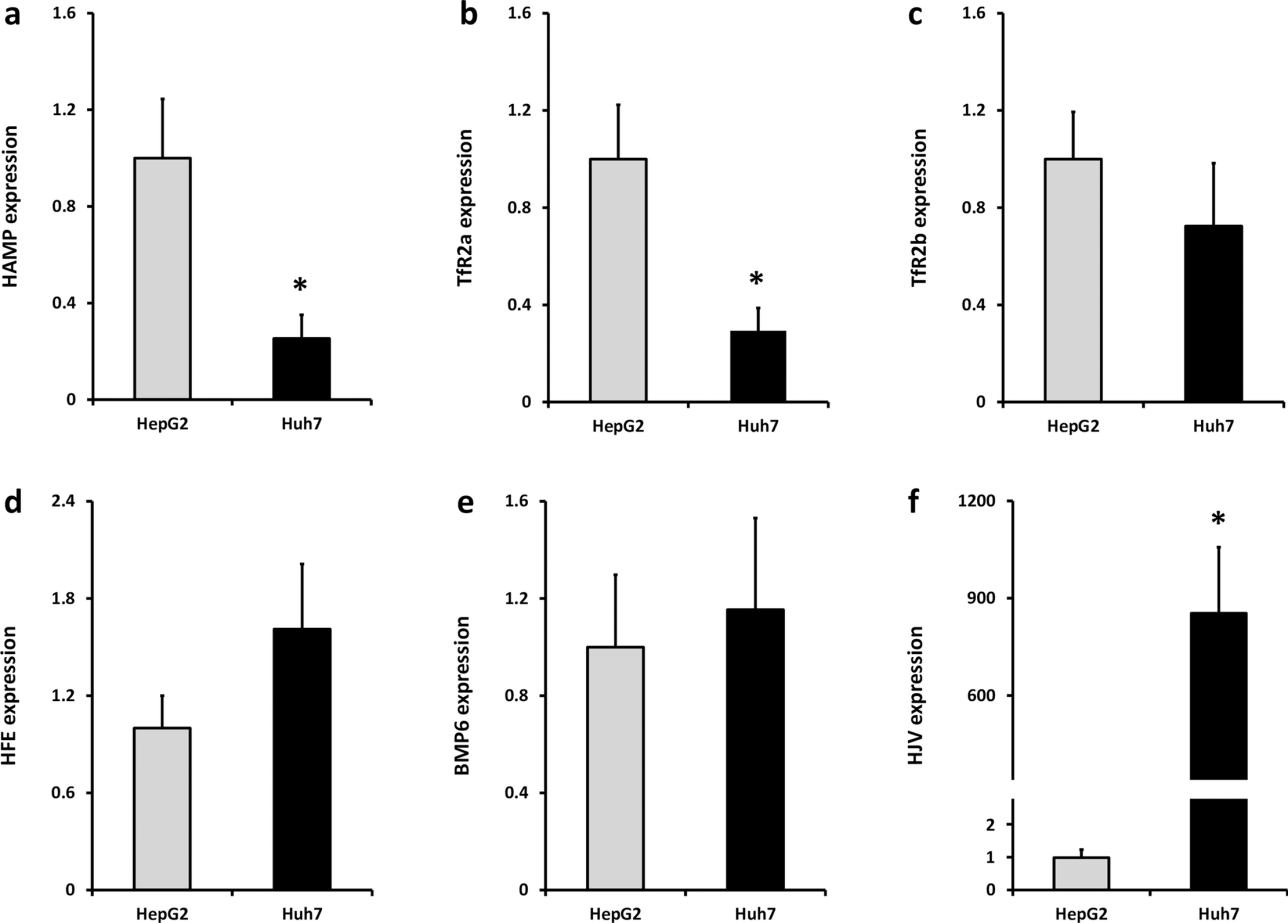
Baseline expression of iron sensing genes in HepG2 and Huh7 cells. mRNA expression of (**a**) *HAMP*, (**b**) *TFR2* alpha, transcript, (**c**) *TFR2* beta transcript, (**d**) *HFE*, (**e**) *BMP6* and (**f**) *HFE2* (*HJV*) were measured by RT-PCR. Data are means ± SEM of 5–8 observations in each group and were normalised to expression in HepG2 cells. * P < 0.02 (Mann-Whitney test).

The relative basal RNA expression levels of iron sensing genes were measured in HepG2 and Huh7 cells, respectively (Fig 1). Expression of *HAMP* and *TFR2* alpha was significantly greater in HepG2 cells compared with Huh7 cells (Fig 1A and 1B). There were no significant differences in levels of *TFR2* beta (Fig 1C), *HFE* (Fig 1D) or *BMP6* (Fig 1E) between the two cell lines. *HFE2* (*HJV*) levels were significantly higher in Huh7 cells (Fig 1F).

We hypothesised that differences in expression may be related to cell-specific methylation of the gene promoters. To investigate this possibility, we interrogated data from the ENCODE project [40] on the University of California Santa Cruz Genome Browser. We restricted our search to CpGs within the 5’ promoter for each gene of interest, since methylation in this region has been show to repress gene expression [38]. At least one CpG was identified in the promoter region of each gene of interest and β-values for the degree of methylation are reported in Table 2. Data were available for experiments with human hepatocytes and with HepG2 cells. In the context of the differences observed in basal expression of *TFR2* alpha and *HAMP* in the hepatic cell lines, it was interesting to note that there was some variation in the degree of methylation of CpGs in both the *TFR2* alpha and *HAMP* gene promoters in hepatocytes and HepG2 cells (Table 2).

**Table 2:** Beta-values from ENCODE database showing degree of methylation of CpG sites in promoters of iron sensing genes.

		Beta-values	
Gene of Interest	CpG identifier	HepG2 cells	Hepatocytes
**TfR2α**	cg10681065	0.09	0.28
	cg04423314	0.09	0.28
**TfR2β**	cg04499151	0.96	0.70
**HFE**	cg06892726	0.08	0.10
	cg05569784	0.21	0.30
**HJV**	cg00953211	0.22	0.13
	cg00987513	0.73	0.27
	cg06589885	0.13	0.05
**BMP6**	cg22505205	0.50	0.13
	cg22541378	0.24	0.03
	cg03447931	0.32	0.02
**HAMP**	cg26283059	0.12	0.27
	cg17907567	0.10	0.25
	cg23677000	0.13	0.21

**Fig 2:**
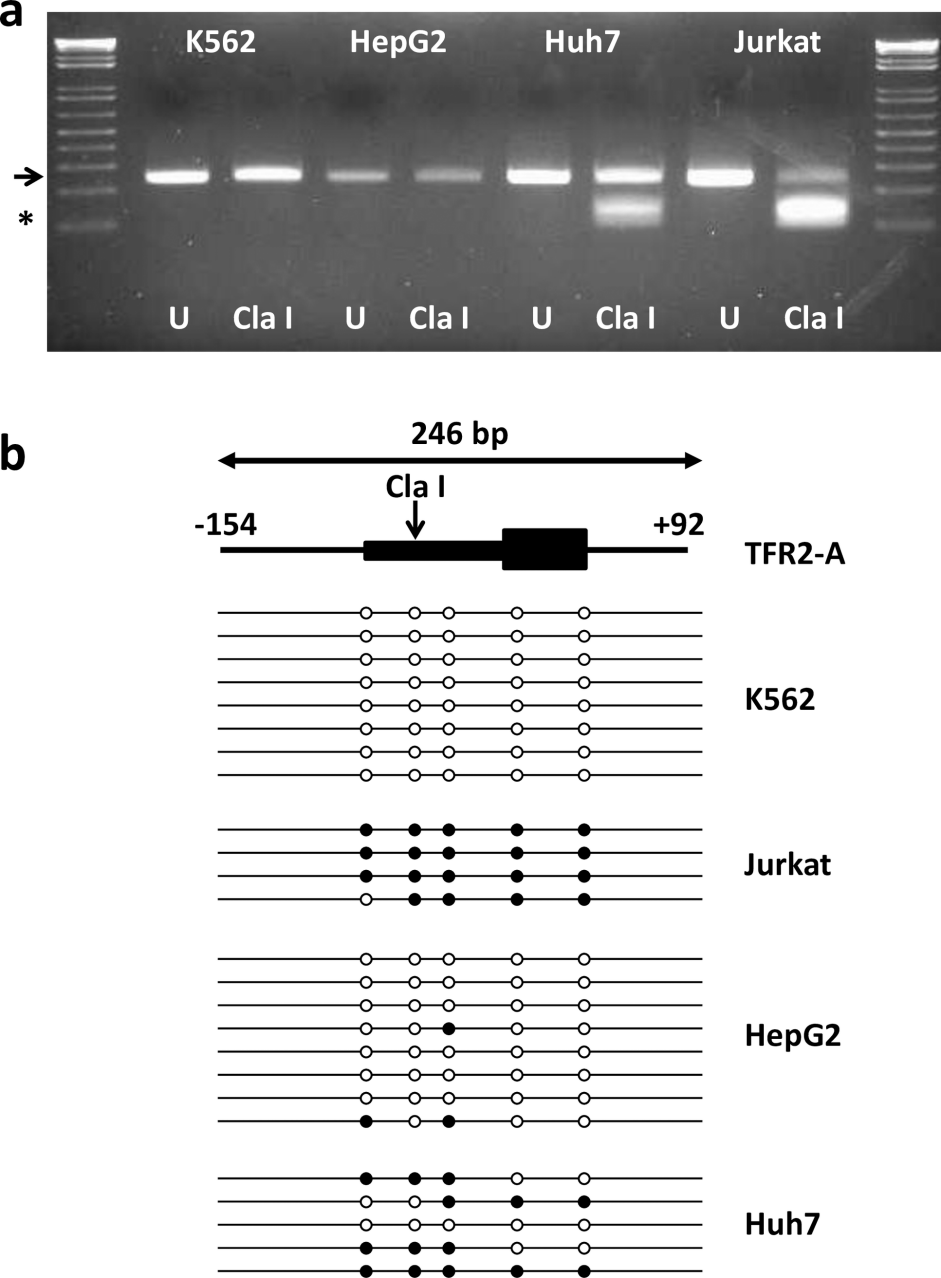
Methylation of *TFR2* alpha promoter in human cell lines. A 246-bp fragment of the human *TFR2* alpha promoter was generated by PCR. Amplicons contained a ClaI restriction digest site which will cut the amplicon into 138 & 108 bp fragments if the CpG within the digest site is methylated. Agarose gel (**a**) shows representative bands from uncut (U; water replacing enzyme) and ClaI-digested bisulphite-converted DNA from K562, HepG2, Huh7 and Jurkat cells. The arrow indicates the position of the full length amplicon (246 bp); * indicates the position of the ClaI digested fragments (138 & 108 bp). The cartoon depicts a representation of *TFR2* alpha gene organization and the 246 bp PCR amplicon (**b**). The 5’ upstream flanking region (from -152 bp relative to the translation start site) and intron 1 (downstream to +92 bp relative to the translation site) are shown as lines; exon 1 containing the promoter and the translated region are shown as boxes. The vertical arrow denotes the ClaI digest site. Horizontal lines below represent bisulphite sequencing of individual amplicons in each cell line. Open circles indicate unmethylated CpGs, filled circles represent methylated CpGs.

To determine whether the *TFR2* alpha and *HAMP* promoters were methylated in Huh7 hepatoma cells, and to confirm their methylation status in HepG2 cells, we carried out bisulphite conversion of DNA and used specific primers to interrogate the CpGs within each promoter that corresponded to the 450K probe sites (Illumina Infinium HumanMethylation450K BeadChip) from the ENCODE project. For *TFR2* alpha, the amplified promoter fragment contained a potential ClaI restriction digest site if the C of the relevant CpG site was retained following bisulphite treatment (i.e., methylated). Following ClaI treatment only the full-length *TFR2* alpha PCR product (246 bp) was identified in HepG2 cells, whereas three fragments (246; 138 and 108 bp) were detected in Huh7 cells (Fig 2A) indicating that the *TFR2* alpha promoter site was unmethylated in HepG2 cells, but partially methylated in Huh7 cells. K562 cells (ENCODE β-values: cg10681065, 0.04; cg04423314, 0.06) and Jurkat cells (ENCODE β-values: cg10681065, 0.88; cg04423314, 0.69) were used as positive and negative methylation controls, respectively. Selection of these cell lines as controls was based on previously published data showing high expression of *TFR2* alpha in K562 cells, but low expression levels in Jurkat cells [12]. Only the full-length *TFR2* alpha PCR product (246 bp) was present in K562 cells, whereas the ClaI digested fragments (138 and 108 bp) predominated in Jurkat cells (Fig 2A). To further investigate the cell-specific difference in *TFR2* alpha promoter methylation, we subjected amplicons from all cell lines to bisulphite sequencing. Five CpG sites were present in the *TFR2* alpha amplicon. All sequenced CpGs in K562 cells and the majority of CpGs in HepG2 cells were unmethylated (Fig 2B). In contrast, 95% of CpG dinucleotides in Jurkat cells and more than 50% of CpGs in Huh7 cells were methylated.

**Fig 3:**
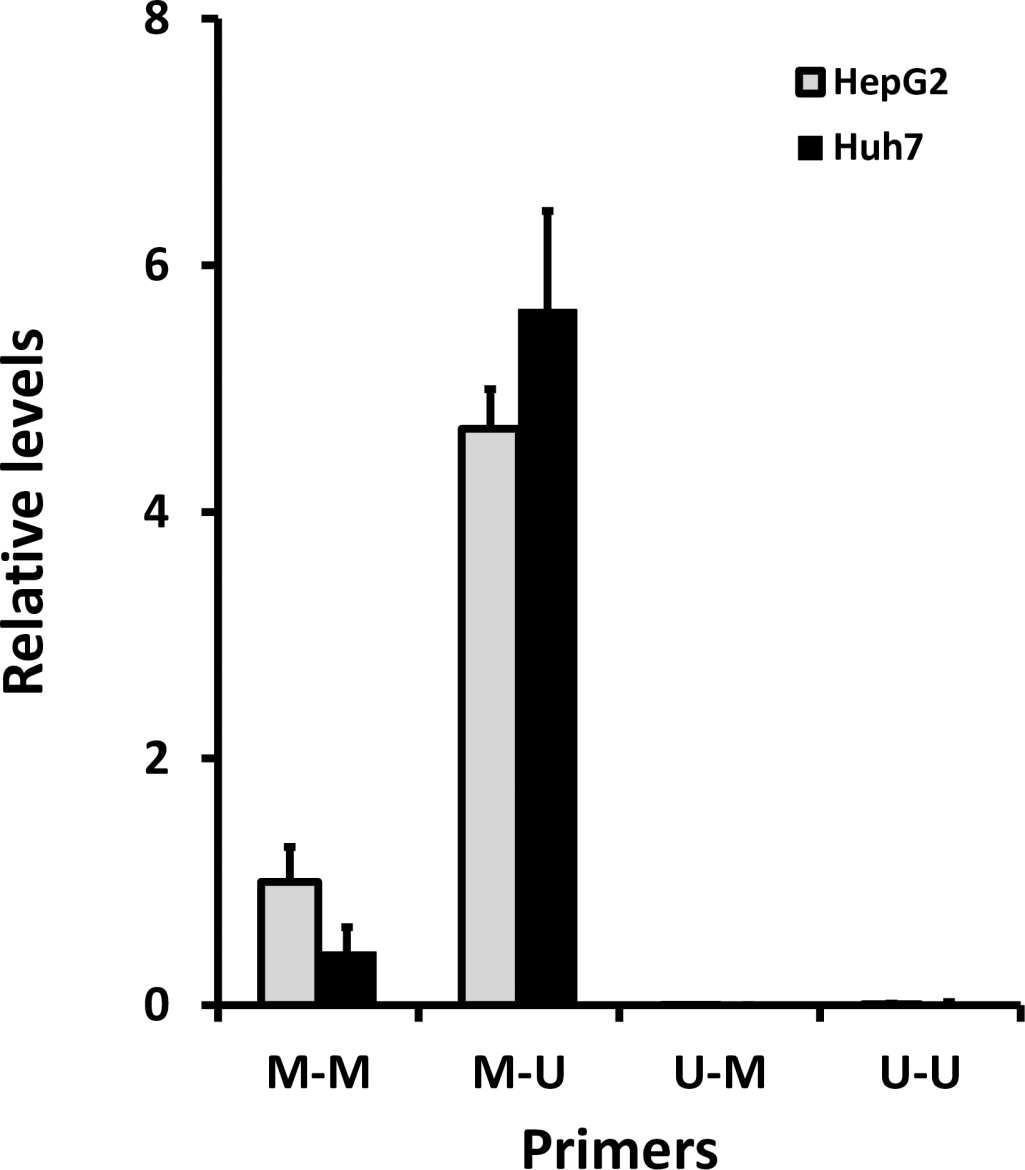
Methylation of *HAMP* promoter. Bisulphite-converted DNA from HepG2 and Huh7 was subjected to qRT-PCR using a combination of primers specific for methylated and unmethylated sequences. Data are presented as relative levels; ΔCt relative to the methylated forward: methylated reverse primer set (M-M) for HepG2 cells.

qRT-PCR analysis of the *HAMP* promoter using combinations of methylated and unmethylated sequence-specific primers revealed no significant differences between HepG2 cells and Huh7 cells for each primer combination (Fig 3). Melt curves indicated only a single PCR product was generated with each of the primer combinations. Lowest Ct values (and therefore highest levels) were observed using the methylated forward: unmethylated reverse primer combination (mean Ct values: 30.6, HepG2; 30.4, Huh7). There was no effect of the demethylating agent 5-deoxy-2’-azacytidine (AZA) on amplicon levels in either cell line using the methylated forward: unmethylated reverse primer combination (HepG2 cells (normalised to control values): control 1.0 ± 0.1; AZA 0.9 ± 0.1; n = 4 for both groups. Huh7 (normalised to control values): control 1.0 ± 0.1; AZA 1.0 ± 0.3; n = 4 for both groups). Since there were no differences in amplicon levels in the presence or absence of AZA in HepG2 and Huh7 cells, we concluded that there was no cell-specific methylation within this region of the *HAMP* promoter and therefore amplicons were not subjected to full bisulphite sequencing.

**Fig 4:**
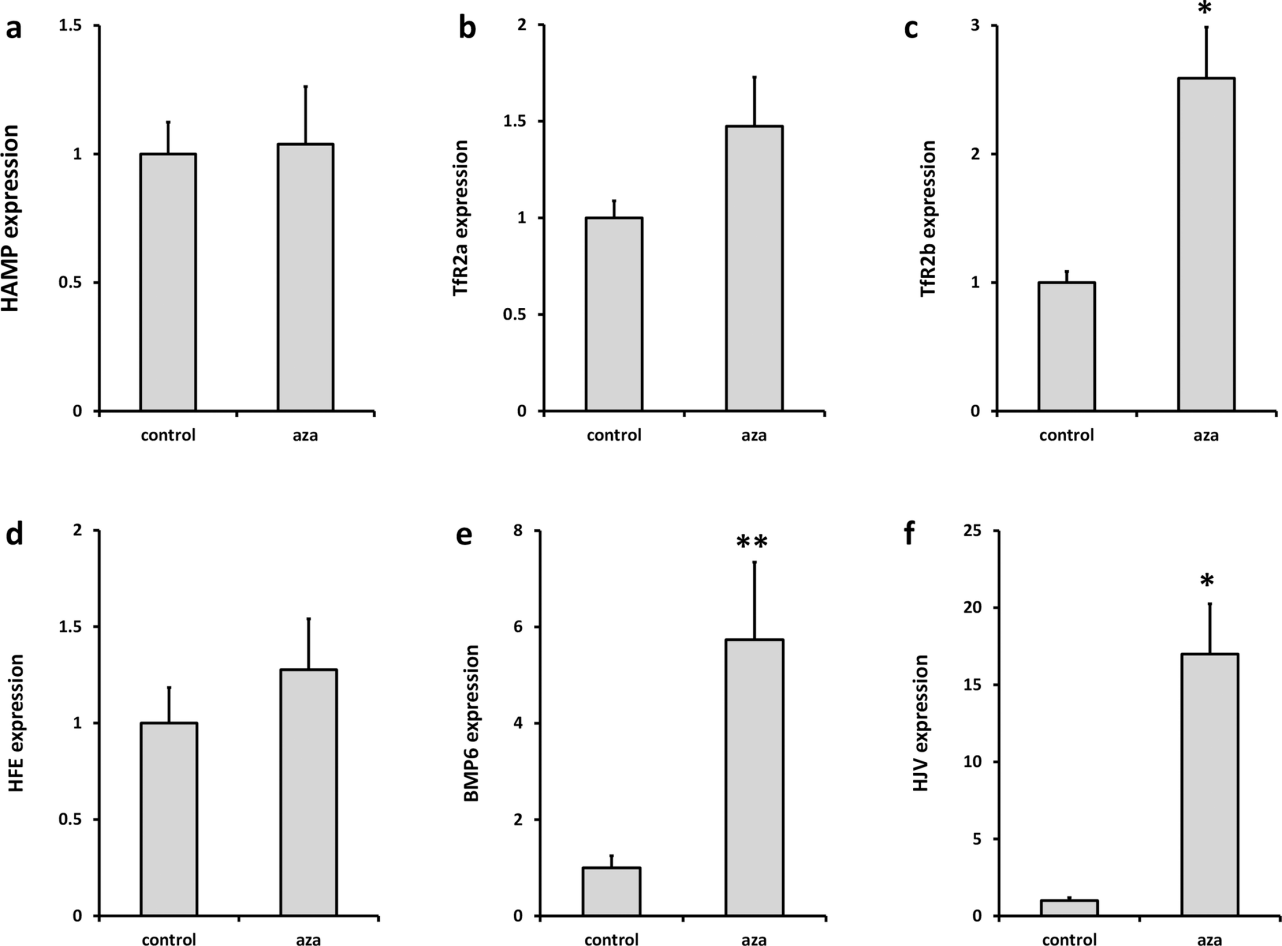
Effect of AZA on iron sensing gene expression in HepG2 cells. Cells were grown in the presence or absence of the global demethylating agent AZA (5 μM, 72 h). Expression of (**a**) *HAMP*, (**b**) *TFR2* alpha, transcript, (**c**) *TFR2* beta transcript, (**d**) *HFE*, (**e**) *BMP6* and (**f**) *HFE2* (*HJV*) were measured by RT-PCR. Data have been normalised to the control group and are presented as mean ± SEM of 7–8 observations in each group. Data were analysed using Mann-Whitney tests. * P < 0.005; ** P < 0.03.

**Fig 5:**
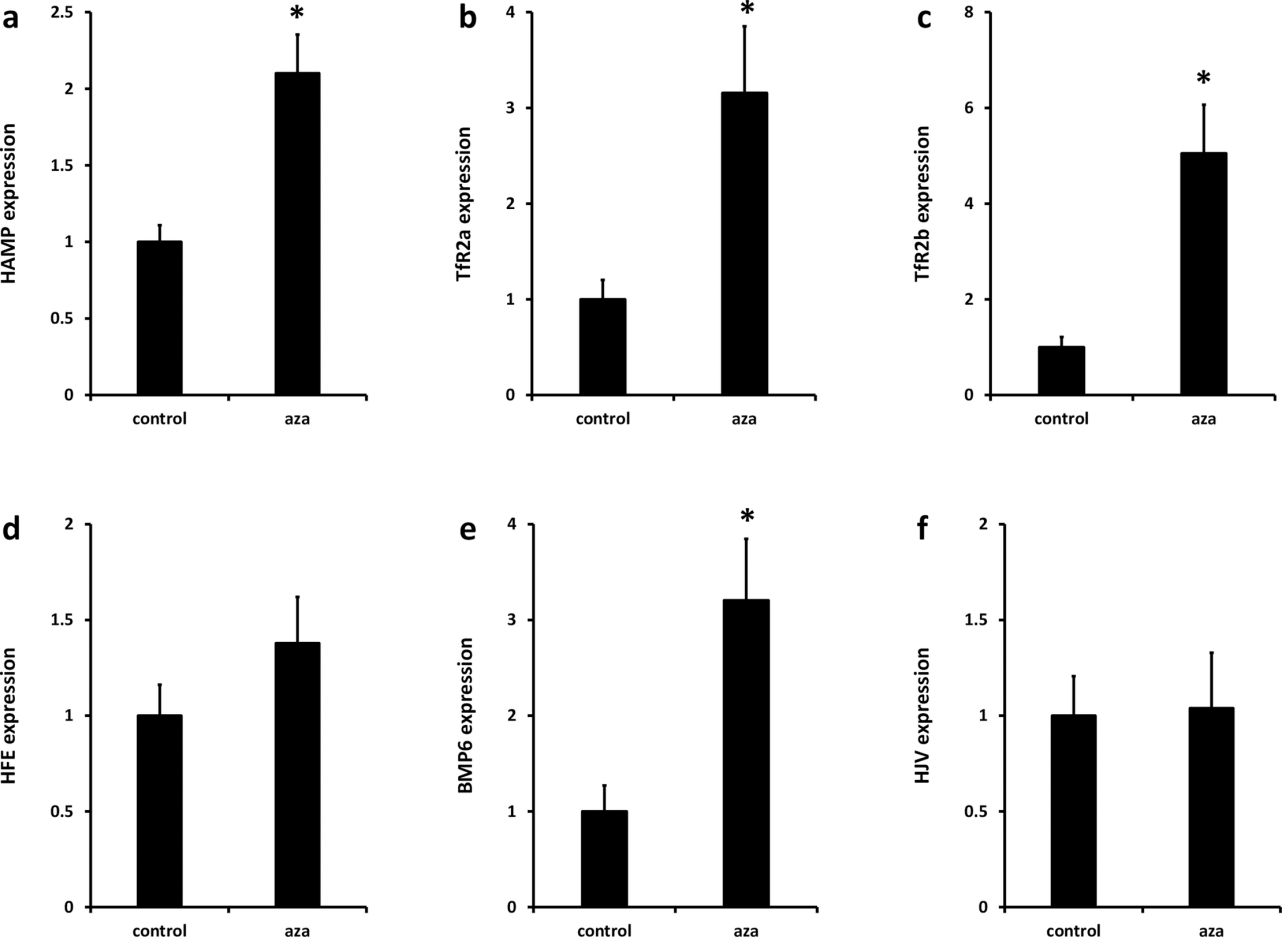
Effect of AZA on iron sensing gene expression in Huh7 cells. Cells were grown in the presence or absence of the global demethylating agent AZA (5 μM, 72 h). Expression of (**a**) *HAMP*, (**b**) *TFR2* alpha, transcript, (**c**) *TFR2* beta transcript, (**d**) *HFE*, (**e**) *BMP6* and (**f**) *HFE2* (*HJV*) were measured by RT-PCR. Data have been normalised to the control group and are presented as mean ± SEM of 9–14 observations in each group. Data were analysed using Mann-Whitney tests. * P < 0.01.

Next we investigated the effects of AZA on the expression of iron sensing genes in HepG2 cells and Huh7 cells. There was no effect of AZA on expression of *HAMP* and *TFR2* alpha in HepG2 cells (Fig 4A and 4B); however, expression of both genes was significantly increased by AZA treatment in Huh7 cells (Fig 5A and 5B). *TFR2* beta (Fig 4C; Fig 5C) and *BMP6* (Fig 4E; Fig 5E) expression was significantly increased by AZA treatment in both cell lines. *HFE2* (*HJV*) expression was increased in AZA-treated HepG2 cells only (Fig 4F; Fig 5F). There was no effect of AZA on *HFE* expression in either cell line (Fig 4D; Fig 5D).

**Fig 6:**
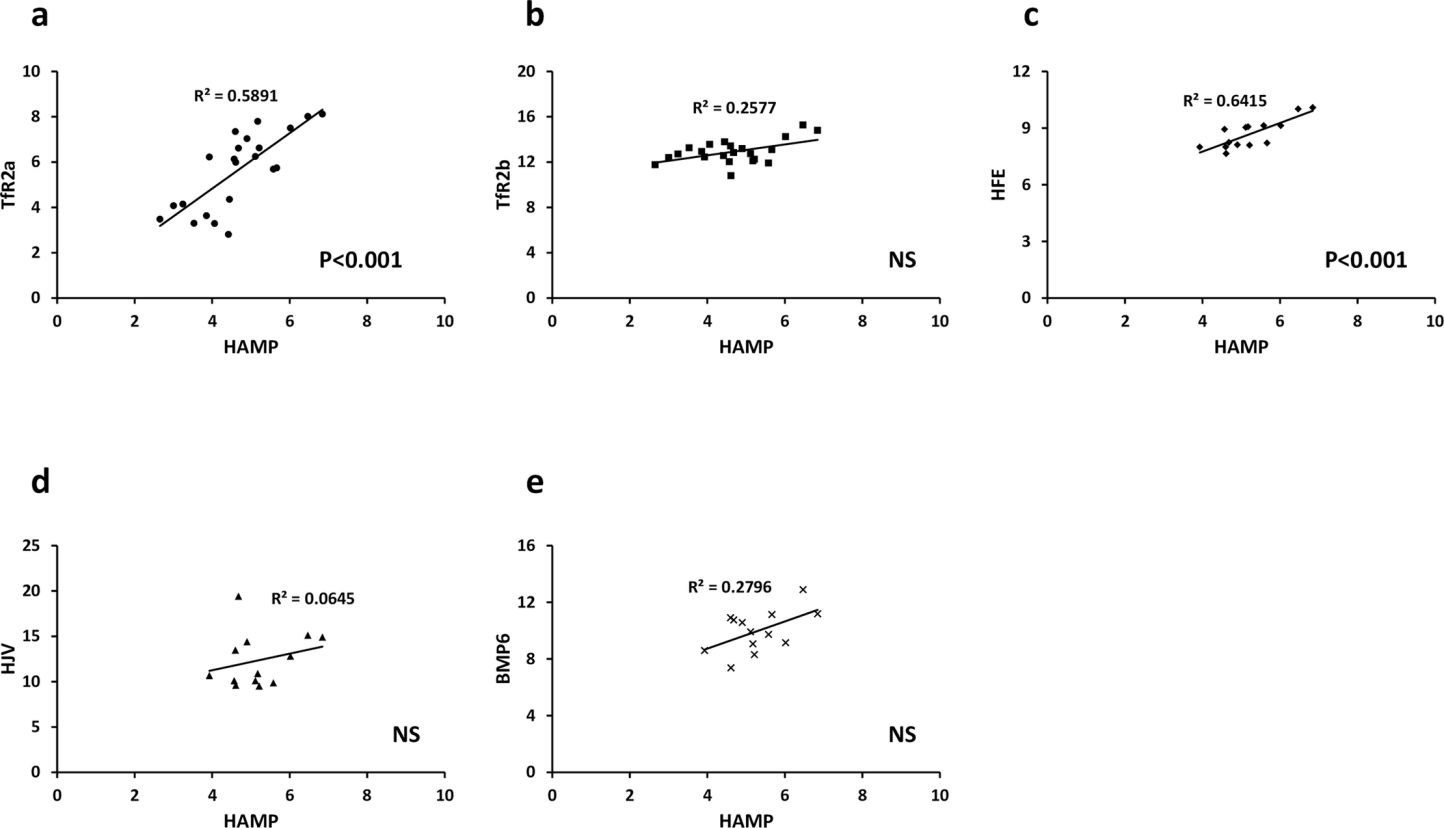
Correlation between iron sensing genes and *HAMP* in HepG2 cells. ΔCt values (using *B2M* as the reference gene) from control and AZA-treated cells were plotted for each gene of interest vs *HAMP*. Data were analysed using linear regression.

**Fig 7:**
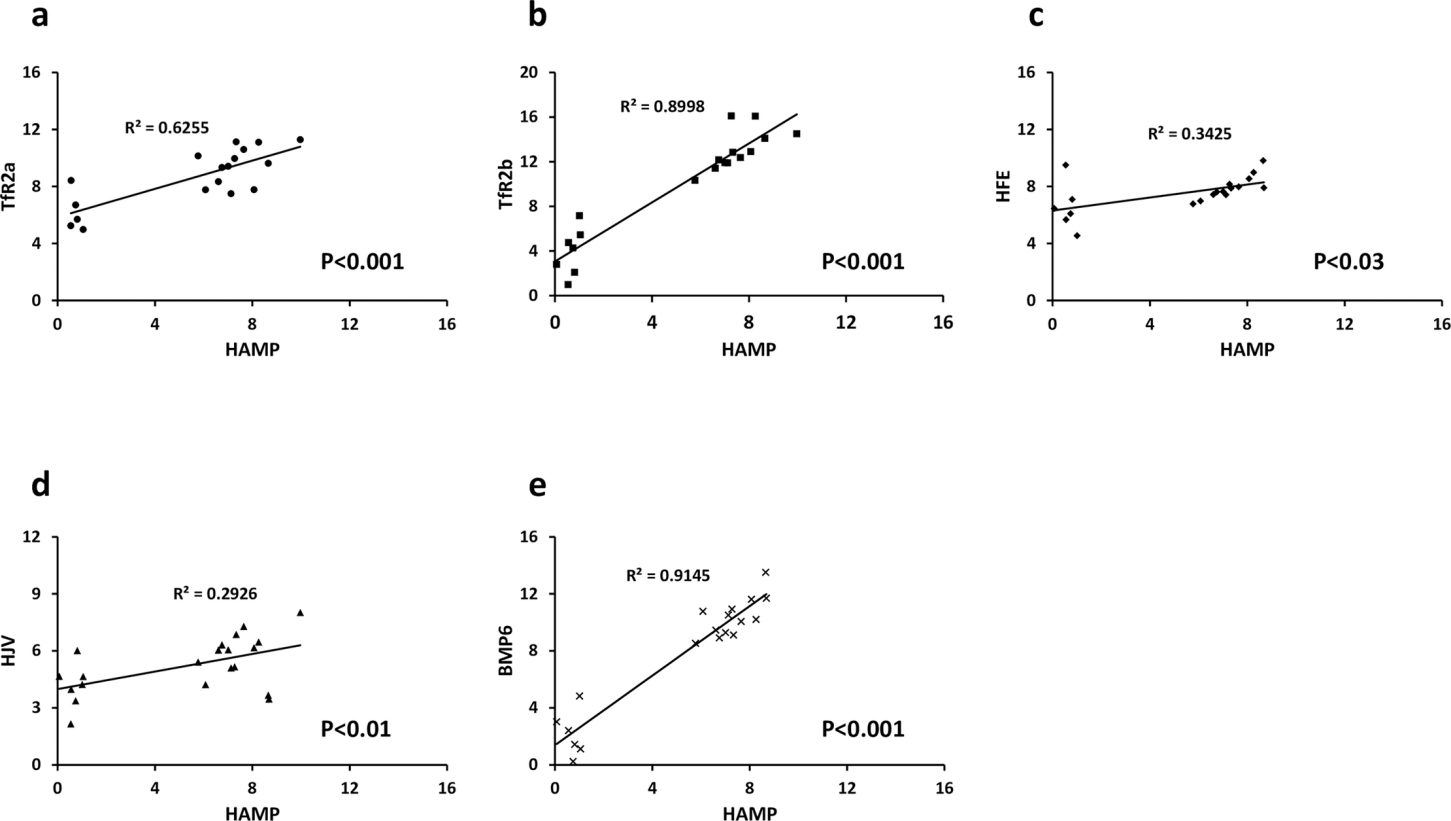
Correlation between iron sensing genes and *HAMP* in Huh7 cells. ΔCt values (using *B2M* as the reference gene) from control and AZA-treated cells were plotted for each gene of interest vs *HAMP*. Data were analysed using linear regression.

Our data indicated that cellular expression and regulation of *HAMP* and *TFR2* alpha followed a similar pattern; i.e., highest expression in HepG2 cells (Fig 1A and 1B) and up-regulation by AZA-treatment only in Huh7 cells (Fig 5A and 5B). However, while there were clear differences in methylation of *TFR2* alpha in HepG2 and Huh7 cells (Fig 2), there was no evidence for cell-specific differences in *HAMP* promoter methylation at documented CpG sites (Fig 3, Table 2). We therefore investigated the possibility that changes in expression of one or more of the iron sensing genes might indirectly influence *HAMP* expression. In HepG2 cells there was significant positive correlation between *HAMP* and *TFR2* alpha, and *HFE* (Fig 6). We carried out multiple linear regression to identify the variables which most significantly predict *HAMP* expression and found that *HFE* was the only significant predictor of *HAMP* mRNA levels (P<0.005). In contrast, in Huh7 cells there was significant correlation between *HAMP* and all iron sensing genes (i.e., *TFR2* alpha, *TFR2* beta, *HFE*, *BMP6* and *HFE2* (*HJV*); Fig 7). Multivariate analysis revealed that *BMP6* (P<0.001) and *HFE2* (*HJV*) (P<0.03) were the only significant predictors of *HAMP* expression in Huh7 cells.

## Discussion

In this study we employed two well-characterised human hepatoma cell lines, HepG2 and Huh7 cells, to investigate the putative effects of methylation on expression of hepatic iron sensing genes. HepG2 cells were originally derived from a well-differentiated hepatocellular carcinoma [41], which displays a high level of morphological and functional differentiation *in vitro*. Huh7 hepatoma cells were derived from a Japanese patient [42] and it has been shown that these cells contain a mutated form of *HFE* [43]. Given that *HFE* is required for iron sensing and appropriate control of *HAMP* expression [17,18], we hypothesized that iron sensing and *HAMP* expression would differ in the two model cell lines.

Basal expression of *TFR2* alpha and *HAMP* was significantly greater in HepG2 cells than Huh7 cells. Differential tissue expression of *TFR2* isoforms has been well documented [12]. *TFR2* alpha is the predominant isoform in liver and blood mononuclear cells, and is highly expressed in associated tumour cell lines, e.g. HepG2 hepatoma cells and K562 erythroleukaemia cells. In contrast, highest expression of *TFR2* beta was observed in spleen and T-cell leukaemia cell lines, e.g., Jurkat cells; these cells also had low expression of *TFR2* alpha [12]. One explanation for the differential expression patterns of the *TFR2* transcripts might be regulation at the level of DNA methylation. To investigate this possibility, we used data from the ENCODE project [40] including the methylation of specific CpG dinucleotides from experiments using the Illumina Infinium HumanMethylation450K BeadChip array platform. We focussed on CpGs in the 5’ promoter of *TFR2*, since methylation of CpGs in gene promoters is associated with silencing of gene expression [38]. For *TFR2* alpha, the methylation pattern from ENCODE (Table 2) and our bisulphite sequencing analysis (Fig 2) showed that CpGs within the *TFR2* alpha promoter were unmethylated in K562 cells and HepG2 cells, but methylated in Jurkat cells. These findings are consistent with previously published mRNA expression data showing high expression of the *TFR2* alpha transcript in K562 and HepG2 cells, but low expression in Jurkat cells [12]. Interestingly, our data demonstrated that the *TFR2* alpha promoter was partially methylated in Huh7, and this was associated with significantly lower basal *TFR2* alpha mRNA expression in Huh7 cells compared with HepG2 (Fig 1B). ENCODE data for HepG2 cells and hepatocytes (Table 2) and our own analysis in Huh7 cells (56% methylation of promoter CpG dinucleotides) found significant methylation of the *TFR2* beta promoter, which may account for low expression of this transcript in both HepG2 and Huh7 cells. Together, we have evidence that differences in DNA methylation are strongly associated with tissue-specific expression of the *TFR2* isoforms.

The tissue-specific epigenetic control of *TFR2* observed here points to tissue-specific functions of the isoforms in the regulation of iron homeostasis. In addition to its well-documented role in hepatic iron sensing, TfR2 is a component of the erythropoietin receptor signalling complex and is required for efficient erythropoiesis [44]. Haematopoietic deletion of *Tfr2* in mice results in impaired erythroid differentiation [45,46]. High expression of *TFR2* in erythroid cells [47] and its role in erythroid differentiation have led to speculation of a clinically relevant role in haematological disorders such as myelodysplastic syndrome (MDS) and acute myeloid leukaemia (AML). In support of this, a recent retrospective analysis of samples from patients with MDS found that expression of *TFR2* alpha and *TFR2* beta was significantly more variable in MDS samples than in non-malignant bone marrow samples. Furthermore, low expression of both *TFR2* transcripts was associated with poorer survival rates in patients with myelodysplastic syndrome with excess blasts than for those with normal to high *TFR2* levels [48]. Similarly, a study in patients with AML found higher expression of both *TFR2* transcripts was associated with significantly longer survival rates [49]. Interestingly, aberrant DNA methylation is the dominant epigenetic alteration in MDS [50] and DNA methyltransferase inhibitors such as azacitidine are approved as treatment strategies in patients with MDS [51].

*HAMP* expression is also known to be tissue specific with highest expression in liver [52] and lower levels present in other tissues including adipose and peripheral blood mononuclear cells [53,54]. ENCODE data suggest that *HAMP* promoter CpG sequences adjacent to the translation start site are unmethylated in HepG2 cells (Table 2), but are partially methylated in both K562 and Jurkat cells (β-scores: cg26283059, 0.31—K562, 0.72—Jurkat; cg17907567, 0.31—K562, 0.45—Jurkat). This is consistent with relatively low expression of *HAMP* in peripheral blood mononuclear cells compared to hepatic cells [54], and may indicate a role for methylation in determining cell- and tissue-specific expression of *HAMP*. To determine whether methylation accounted for the observed differences in basal expression of *HAMP* between HepG2 and Huh7 cells we analysed the region of the promoter containing the CpG dinucleotides cg17907567, cg23677000 and cg26283059. These CpGs are unmethylated in HepG2 cells (Table 2) and our studies suggest that there is no difference in methylation between HepG2 and Huh7 cells. In summary, our analysis does not support the hypothesis that differential methylation of this region of the *HAMP* promoter explains the differences in basal expression of *HAMP* between HepG2 and Huh7 cells.

To assess the effects of methylation on *HAMP* and *TFR2* alpha mRNA expression we treated cells with the 5-aza-2’deoxycitidine (AZA), which inhibits DNA methyltransferase activity and results in global DNA demethylation [55]. AZA treatment increased *TFR2* alpha mRNA expression in Huh7 cells, but not in HepG2 cells. Taken together, our data and those from ENCODE suggest that differential expression of *TFR2* alpha between HepG2 cells and Huh7 cells is mediated by methylation of promoter CpG dinucleotides. Furthermore, this is also a likely explanation for tissue-specific expression of TfR2 isoforms demonstrated previously [12]. Expression of *TFR2* beta and *BMP6* was increased in both HepG2 and Huh7 cells, and *HFE2* (*HJV*) was elevated in HepG2 cells following AZA treatment indicating that methylation plays an important role in regulating the expression of a number of iron sensing genes.

AZA treatment significantly increased *HAMP* expression in Huh7 cells, but did not alter *HAMP* levels in HepG2 cells. Interestingly, a recent report has shown that *HAMP* expression is supressed in hepatocellular carcinoma through hypermethylation of CpGs within the gene promoter [56]. These findings are in contrast to the data presented here. However, it is important to note that the *HAMP* promoter region analysed by Udali et al. (-940 to -398 bp relative to the translation start site) [56], did not overlap with the promoter amplicon studied in our analysis (-186 to +14 bp relative to the translation start site). It is possible therefore that hypermethylation within the distal promoter modulates *HAMP* expression. Moreover, it is possible that epigenetic silencing of elements within one or more of the iron signalling pathways may contribute to decreased *HAMP* expression. For example, SOSTDC1, an inhibitor of BMP 2, 4 and 7 activity, decreases SMAD-signalling and subsequent hepcidin secretion in prostate epithelial cells [57]. Interestingly in prostate cancer the *SOSTDC1* gene promoter is highly methylated leading to suppression of gene transcription, and this is associated with increased *HAMP* expression and poorer prognosis in patients with prostate tumours [57].

A further possibility is that differential methylation of one or more of the hepatic iron sensing genes could have an indirect effect on *HAMP* expression. Our previous work shows that *TFR2* mRNA correlates significantly with *HAMP* expression levels in human primary hepatocytes [58], while others have shown strong correlation between *HFE* and *HAMP* in HepG2 cells [59]. Here we found a significant correlation between levels of both *TFR2* alpha and *HFE*, and *HAMP* in HepG2 cells, with *HFE* being the most significant predictor of *HAMP* expression. Interestingly, while there is significant positive correlation between *HAMP* and each individual iron sensing gene in Huh7 cells, multivariate analysis showed *HFE2* (*HJV*) and *BMP6* to be the only significant predictors of *HAMP*. This divergence between the iron sensing pathways that correlate with *HAMP* expression in HepG2 and Huh7 cells is perhaps not surprising given that there is a mutation in the *HFE* gene in Huh7 cells [43], which alters the conformation of the α3 domain of the mature protein. TfR2 interacts with the HFE α3 domain [60], and therefore this mutation might silence downstream signalling pathways in Huh7 cells which regulate *HAMP* expression. Similarly, the very low basal expression of *HFE2* (*HJV*) in HepG2 cells, due at least in part to DNA methylation, is likely to dampen BMP/SMAD signalling in these cells. Interestingly, in patients with MDS, the *HFE2* promoter in bone marrow cells was found to be hypermethylated. Treatment with AZA increased *HFE2* expression and this was associated with elevated serum hepcidin levels [61]. Taken together, our data suggest that HFE/TfR2 signalling predominates as the iron sensing pathway in HepG2 cells, while the BMP/HJV/SMAD pathway is dominant in Huh7 cells. Key iron sensing genes in both cell lines may be silenced by DNA methylation and this may have an important bearing on *HAMP* expression.

In summary, our study provides evidence for a role of DNA methylation in controlling hepatic iron sensing and the production of hepcidin. Serum hepcidin levels in the healthy population are highly heterogeneous [35–37] and differential patterns of DNA methylation may be important in determining the variability in iron status and hepcidin production at a population level. This possibility remains to be explored. It has been demonstrated recently that there is widespread inter-individual epigenetic variation, including DNA methylation, in human neutrophils from healthy individuals, which might relate to phenotypic differences and potentially susceptibility to a range of diseases [62]. The epigenetic influences on iron homeostasis and the risk of developing iron metabolism disorders may be similarly linked and this possibility warrants further investigation.
